# LiftingWiSe: A Lifting-Based Efficient Data Processing Technique in Wireless Sensor Networks

**DOI:** 10.3390/s140814567

**Published:** 2014-08-11

**Authors:** Emad Aboelela

**Affiliations:** Computer Engineering Department, College of Computer Science and Engineering, Taibah University, Madinah 41477, Saudi Arabia; E-Mail: eaboelela@taibahu.edu.sa; Tel.: +966-535-813-263

**Keywords:** wireless sensor network (WSN), data dissemination, data compression, data aggregation, objects monitoring, internet of things (IoT)

## Abstract

Monitoring thousands of objects which are deployed over large-hard-to-reach areas, is an important application of the wireless sensor networks (WSNs). Such an application requires disseminating a large amount of data within the WSN. This data includes, but is not limited to, the object's location and the environment conditions at that location. WSNs require efficient data processing and dissemination processes due to the limited storage, processing power, and energy available in the WSN nodes. The aim of this paper is to propose a data processing technique that can work under constrained storage, processing, and energy resource conditions. The proposed technique utilizes the lifting procedure in processing the disseminated data. Lifting is usually used in discrete wavelet transform (DWT) operations. The proposed technique is referred to as LiftingWiSe, which stands for Lifting-based efficient data processing technique for Wireless Sensor Networks. LiftingWiSe has been tested and compared to other relevant techniques from the literature. The test has been conducted via a simulation of the monitored field and the deployed wireless sensor network nodes. The simulation results have been analyzed and discussed.

## 1. Introduction

The recent rapid advancement in the wireless sensor network field has encouraged researchers to work towards building a comprehensive physical world networked with sensors. Such a network is referred to as a Wireless Sensor Network (WSN). One of the potential applications of WSNs is the real-time monitoring of objects. Each of the monitored objects is associated with a WSN node. This is particularly important if the objects are deployed over large hard-to-reach areas. However, such an application requires disseminating real-time data about each node through the WSN in a mesh-networking scheme. The data is usually disseminated towards the network sink node. The sink node acts as the gateway of the WSN to the backbone data network (e.g., the Internet). Such data dissemination processes have to be efficient due to the energy, processing, and memory constraints of the WSN nodes. The batteries of WSN nodes are usually small and hard to replace once deployed in the monitored field. One way for energy conservation in the WSN is to reduce the amount of data transmission and disseminate this data towards the sink using the shortest paths. Generally, radio communication among the WSN nodes consumes much more power than data processing within the nodes [1]. Reducing data transmission can be achieved through applying efficient data aggregation or compression techniques within the WSN nodes [2].

In this paper, I discuss the processes of compressing and disseminating data in WSNs. For the compression process, I studied three simple compression techniques that compress the disseminated data within the WSN. Simplicity of such techniques is needed as this study focuses on monitoring a large number of WSN nodes, which are constrained by very limited processing and memory resources. The first technique is a compression technique based on calculating the offset of the transmitted data (the signal) from a reference value. The second scheme is an algorithm explained in [3], which is based on the Huffman coding algorithm [4]. The third scheme is a proposed compression scheme, which is based mainly on the discrete wavelet transform (DWT) via a lifting procedure [5,6]. The DWT has been applied in many disciplines such as computer science, engineering, and mathematics. Its most important applications are in the fields of digital signal processing and data compression (e.g., JPEG 2000) [7].

The compression techniques were simulated and tested under different scenarios of data dissemination within a WSN. The simulation results show that the proposed lifting-based technique, called LiftingWiSe, outperforms the other tested techniques in terms of the compression ratio and the needed memory to store the received/processed/transmitted monitoring data within the WSN nodes.

The rest of the paper is organized as follows: Section 2 presents work related to the proposed technique. In Section 3, the compression schemes used in the simulation are described. The methodology used in testing the compression techniques is summarized in Section 4. The experimental results and analysis are covered in Section 5. Finally, Section 6 concludes the paper.

## 2. Related Work

Both industry and academia have recently focused on projects that utilize WSNs in monitoring applications. Such projects include transmission of large amounts of data (e.g., video) that covers public or hard-to-reach venues [8,9]. The main challenge in implementing these projects is how to meet the service performance and cost requirements that are limited by the energy and processing constraints of the WSN nodes. A variety of power management strategies have been proposed to save WSN nodes' energy, such as providing different power modes in the nodes. These include the on, ready, doze, sleep, idle, and hibernate modes [8]. Another strategy is to use power-efficient sensors and radio hardware. However, radio communication consumes much more power than data processing within the nodes [1]. Therefore, strategies are needed for bandwidth efficiency such as modified communication protocols and in-network data processing [9].

Semantic clustering is an example of such modified communication protocols for WSNs. In semantic clustering the WSN is divided into a number of clusters (group of nodes) formed by semantically related nodes. At least one node in each cluster is elected to be the semantic collector. The semantic collector acts as a bridge between the cluster internal nodes and the WSN sink node. In recent research studies, semantic clustering models based on fuzzy inference have been proposed [10,11]. In [11] the proposed algorithm elects the semantic collectors based on the nodes' residual energy level and their received signal strength indicator to the sink.

Data aggregation, compression, and prediction are examples of the in-network data processing approaches to handle the limited resources in the WSN nodes [12]. They have received significant attention from the researchers within the WSN field [2,13]. The main goal of the proposed techniques in the literature is to reduce the size of the transmitted packets in the WSN and hence increase the lifetime of the network. Another goal is to reduce the memory needed to store the received/processed/transmitted data within the WSN nodes. Some techniques are based on simple aggregation functions such as average, minimum, and maximum [14,15]. Other techniques are based on spatial or temporal correlation to predict some data without the need to propagate this data all the way to the sink [16]. These techniques are not appropriate for the monitoring problem presented in this paper. Monitoring objects requires collecting data about each of the monitored objects such as their locations. Such data can neither be aggregated nor be predicted to avoid loss or inaccuracy of data. Therefore, the suitable option for the monitoring problem is to compress the transmitted data. However, not all compression techniques are feasible for WSNs [12]. This is due to the limited processing capability and memory space in the WSN nodes. In the remainder of this section, I will summarize some studied compression techniques that are suitable for WSN applications.

Different data compression techniques that are suitable for WSN have been surveyed [13,17]. Kimura and Latifi [13] presented four categories of those compression techniques: coding by ordering, pipelined in-network compression, low-complexity video compression, and distributed compression. Coding by order is utilized within the data funneling routing technique proposed in [18]. In that routing technique, some of the sensor nodes work as a data aggregation node. The pipelined in-network compression technique trades high data transmission latency for low transmission energy consumption [19]. Aggregation nodes collect sensor data in their buffers for a certain amount of time. During that time, data packets are combined into one packet after removing any redundancies in the collected packets. The low complexity video compression scheme is based on block changing detection algorithm and JPEG data compression [20]. The distributed compression technique utilizes side information to encode correlated source information [21]. The rationale behind this technique is that WSN nodes are usually densely populated in the deployment field and hence such correlation conditions can be easily satisfied.

In a more recent survey, Razzaque *et al.* [17] presented a logical classification of existing WSN data compression approaches. They indicated that each WSN data compression technique belongs to one of the following categories: aggregation, text-based compression, distributed source coding, transform-based compression, compressive sensing, or predictive coding. Analysis of the performance and efficiency of each approach was presented in the survey. They showed that the aggregation technique is easy to deploy, but it has limited applications because the original data cannot be reproduced at the sink node. Predictive coding has a high compression ratio. However, it has a significant overhead of learning data statistics. Distributed source coding has the overhead of obtaining the correlation knowledge, which makes this technique suffer in dynamic WSN environments. Transform-based compression and compressive sensing both do not require learning of correlation statistics and hence they are effective in dynamic WSN environments. Transform-based compression techniques are useful for multimedia communications over WSNs. The study in [17] concluded that still more issues need to be resolved in the research field of data compression in WSNs. These unresolved issues include the scalability and reliability of the proposed compression techniques.

Sadler and Martonosi [22] proposed a data compression algorithm for energy-constrained devices. It is a dictionary-based lossless compression algorithm. They call their algorithm S-LZW, which stands for a sensor version of the LZW algorithm [23,24]. They tailored the algorithm to meet the energy and memory constraints of sensor nodes.

Marcelloni and Vecchio [3] presented a simple WSN compression algorithm. This algorithm follows a scheme similar to the one used by the baseline JPEG algorithm. The algorithm was compared with the S-LZW algorithm [22]. The results showed that the presented simple algorithm outperforms S-LZW in terms of compression ratio. The algorithm utilized by [3] was the Huffman's coding.

Pudlewski *et al.* [25] introduced a wireless video transmission system based on compressed sensing. They applied the compressed sensing (CS) theory for the compression component of the system. The simulation results showed that the system is suitable for wireless multimedia sensor networks (WMSN). WMSNs are used to acquire, process, correlate, and fuse multimedia streams in a distributive and real-time fashion. WMSNs are suitable for applications such as video surveillance and objects locator services.

The DWT proved remarkably successful in data compression (e.g., JPEG 2000) [5,6]. Recent research efforts have studied different approaches of utilizing wavelet transforms in WSNs' data dissemination. A tutorial in [26] presented DWT-based techniques for in-network compression that can be used in WSN nodes with very small random access memory (RAM). The focus is on image processing where a small camera is directly connected to the microcontroller of the sensor nodes. In the tutorial the authors underlined that even though the presented techniques are not lossless, the evaluations illustrated that the loss is typically not visible.

Ciancio and Ortega [27] proposed a distributed compression algorithm for multi-hop sensor networks based on the lifting factorization of the wavelet transform. The proposed algorithm utilizes the data flow direction in the network to perform partial wavelet coefficients computations at each sensor node. The algorithm was tested on small networks (10–100 nodes) with a pre-organized distribution of the sensor nodes that are placed linearly in 1-D (one-dimensional) paths. Simulation results showed that the transmission cost depends on the network configuration. Ciancio *et al.* [28] proposed an extension to their work by considering 2-D (two-dimensional) network, which they treated as a combination of multiple 1-D paths. They incorporated routing algorithms with their data compression algorithm. Based on the network topology, each sensor node is allowed to select the coding method that suitable for it. The authors compared multiple routing techniques to identify the most efficient one for the topology at hand. The analysis was limited to predefined routing topologies on a small network of 100 nodes.

A DWT-based energy-efficient audio compression scheme for wireless multimedia sensor network (WMSN) was proposed in [29]. The authors considered the WSN nodes were deployed uniformly in a 2-D grid with a fixed distance between any two adjacent nodes. The sink node was placed in the center of the grid. They used DWT to capture spatial and temporal correlation among the disseminated data in the WSN. The proposed scheme was tested on a simulation of 256 nodes.

Wagner *et al.* [30] studied the application of DWT for a 2-D irregular sensor node placement. By irregular placement, they mean a non-grid placement of the nodes. Each node needs to determine its location in the deployment field through a proposed distributed triangulation algorithm. The proposed technique requires the construction of a distributed mesh among the nodes in the network. A significant communication overhead is needed to maintain that mesh. The proposed technique was tested by creating 300 sample points from uniformly distributed random node locations. Wagner *et al.* [31] extended the work in [30] by proposing a meshless solution to the problem. The techniques proposed in [30,31] require backward data transmissions that flow away from the sink, which adds a communication overhead.

In this paper, the focus is on a WSN model where thousands of objects need to be monitored. Both random and grid deployment of the sensor nodes were simulated. A modified version of the lifting factorization of the wavelet transform is proposed. Nodes in the deployment field do not need to know their location in the deployment field. Each node needs to communicate with its neighbors over an arbitrary routing tree. Specifically, a node communicates with the nodes that are closer to the sink (root) node of the routing tree. The proposed technique performs the wavelet transform as data flows towards the sink. There is no need for backward data transmission from the sink. According to the classifications of the WSN compression techniques presented in [17], the proposed technique can be classified as data, communication, scalable, real-time, and on-route compression. The performance of the proposed technique, LiftingWiSe, is compared with the algorithm presented in [3].

## 3. Compression Schemes

Three compression schemes are compared in this study. These compression schemes are the Offset, Marcelloni, and the proposed LiftingWiSe technique. The tested schemes have a common feature of being simple to process within a WSN node. This is an essential feature as the focus in this study is on WSNs with large numbers of nodes and with very limited processing capability and memory space. In this paper, compression is applied on each sequence of related data individually. Throughout the rest of this paper, I will refer to the “sequence of related data” as a *signal*.

In the monitoring system at hand, each sensor node handles three signals: the first one carries the stimulus value at the location of the sensor node. The location is represented by the second and third signals, which carry the sensor nodes' X and Y coordinates. The stimulus represents any environmental condition such as the temperature, the humidity, or the pressure. Compression schemes can be classified as lossless or lossy compression. A *lossless compression* scheme compresses a signal of data but never discards any details in the original signal. It allows the exact original data to be reconstructed from the compressed data. A *lossy compression* scheme compresses a signal of data by discarding (losing) some tiny details in the original signal with the goal of increasing the compression percentage. The location coordinates and stimulus values can be compressed using either lossy or lossless schemes as they afford a slight change in their disseminated values. In the following subsections, I will explain how the Offset, Marcelloni, and the proposed LiftingWiSe compression schemes are implemented.

### 3.1. Offset Compression

The minimum absolute value in the signal is used as a “reference”. Each value in the signal is replaced by the difference between the value and the reference. The node disseminates the compressed signal that contains the differences along with the reference value. The idea of the Offset scheme is to have a signal with small numbers, which require fewer bits to encode and transmit.

### 3.2. Marcelloni Compression

The Marcelloni compression scheme is based upon the algorithm proposed in [3]. The algorithm is based on Huffman's coding. Huffman coding maps a sequence of symbols to bits of variable sizes, so that symbols appear more frequently are represented by a smaller number of bits than those that occur rarely.

The Marcelloni algorithm replaces each entry in the signal, *r_i_*, with the difference *d_i_*, where *d_i_* = *r_i_* − *r_i_*_−_*_1_*. Each *d_i_* is encoded using two parts *s_i_*|*a_i_*. Given *n_i_* as the number of bits needed to encode *d_i_* then *s_i_* is the Huffman coding of *n_i_*. Therefore, *s_i_* is a variable-length binary code generated by using Huffman coding on *n_i_*. Finally, *a_i_* is the binary representation of *di*. Special cases of the entries in the generated signal are explained by Marcelloni and Vecchio [3]. In these special cases the variable-length integer code part of the bit sequence, *a_i_*, is represented based on the value of *d_i_*. Different representation are used for the following ranges of *d_i_*: *d_i_* > 0, *d_i_* < 0, and *d_i_* = 0. The presented Marcelloni compression technique is lossless. It was tested by [13] on temperature and relative humidity signals.

### 3.3. LiftingWiSe

The proposed compression technique is an arbitrary length compression scheme that is based mainly on the DWT via lifting procedure [5,6]. The lifting procedure can be applied on a signal *S_j_* of length 2*^j^*, where *j* is a positive integer. Each lifting round consists of three steps [5,32]:

(1) *Split*: The signal *S_j_* is transformed into two sequences, the odd sequence *d_j_*_−_*_1_* and the even sequence *s_j_*_−_*_1_*, each of length 2*^j^*^−1^. *Note:* in the implementation of this step, the split is done logically on the same physical array of the original signal.
(2) *Prediction*: It represents the correlation between an entry in the signal and its nearest neighbors. In this paper, the difference between the odd indexed entries in the signal and its preceding even indexed entries is used for the prediction step. Jensen and la Cour-Harbo [32] used the prediction step to calculate the entries of the *d_j_*_−_*_1_* sequence as in Equation (1):
  (1) $$d_{j-1}\left[\text{k}\right]=s_{j}\left[\text{2k}+\text{1}\right]-s_{j}[\text{2k}],\;\text{where}\;\text{1}<\text{k}<{\text{2}}^{\text{j}-\text{1}}\;$$
(3) *Update*: Here the even entries of the signal (*i.e.*, *s_j_*_−_*_1_*) is replaced with the average of the original even and odd entries as in Equation (2):
  (2) $$s_{j-1}[\text{k}]=s_{j}\left[\text{2k}\right]-d_{j-1}[\text{k}]/\text{2},\text{where}\;\text{1}<\text{k}<{\text{2}}^{\text{j}-\text{1}}$$

**Figure 1.**
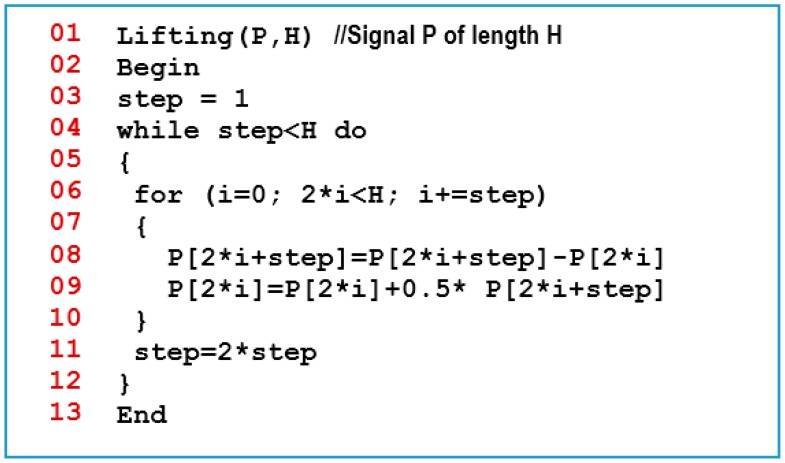
The Lifting compression algorithm.

The proposed LiftingWiSe technique is a modified version of the original lifting procedure. It can be applied on a signal with an arbitrary length. This has been achieved by first dividing the signal *S* of any positive-integer length *L* into two parts. The first part, *S_n_*, contains the first 2*^n^* entries in *S*, where *n* = *log*_2_*L*. The second part, *S_r_*, contains the remaining entries in *S*. The lifting algorithm shown in Figure 1 is applied on *S_n_*.

In the shown lifting algorithm, the code in line 8 represents the “prediction” procedure while the code in line 9 represents the “update” procedure. The same procedures, prediction and update, are repeated on the generated updated signal recursively until the generated signal has only one entry. The final resulted signal combines all the signals generated from the prediction procedures. It contains values with smaller dynamic range than the original signal. This potentially leads to a better compression. One more thing to note is that the first entry in the resulted signal is actually the mean value of all entries in the original signal [7].

**Figure 2.**
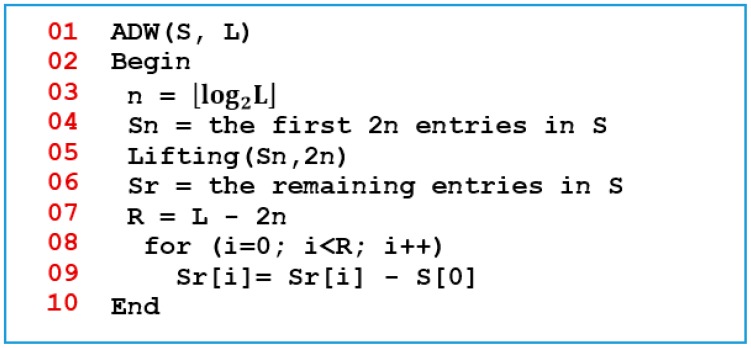
The Proposed Compression Technique.

Figure 2 shows the pseudo code of the proposed LiftingWiSe compression technique where the above lifting algorithm is applied on *S_n_*. The first entry in the result, the mean value, is subtracted from all entries in *S_r_*. This way; the proposed technique can be applied on signals with an arbitrary length. The LiftingWiSe technique is not lossless due to the division by 2 operation in step 9 of Figure 2. The result of that operation has to be rounded as the signal contents are restricted to be integers. However, the rounding operation usually compensates the loss at one stage with a gain in a following stage and vice versa. The results show that the loss in the signals is negligible. Generally, the low-memory wavelet transform techniques are not lossless. However, studies illustrated that the loss is typically not visible [26].

**Figure 3.**
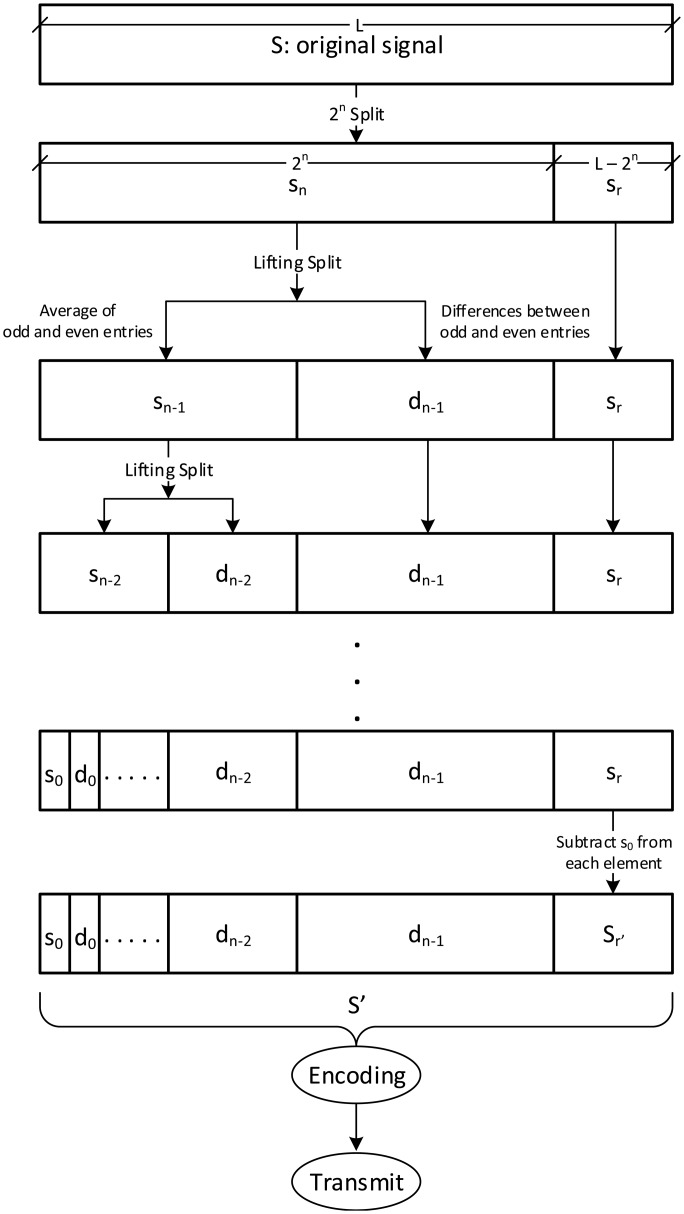
LiftingWiSe signal processing chart.

Figure 3 shows the LiftingWiSe signal processing chart where the above algorithms are applied on signal *S* to produce the processed signal *S′*. An important benefit of the presented process is that throughout the process, the operations are performed in place. This means the entries in the signal are replaced with the predicted, updated, and difference entries without the need for extra temporary storage to carry out those operations. This is especially important for the wireless sensor nodes where memory space is limited.

During the encoding stage, the entries of the processed signal, *S′*, are converted into binary words. To avoid the effect of noises, two word sizes are used to encode *S′*. Equation (3) shows the calculations of the word sizes, *W*, used to encode the entries in *S′*. The smaller word size fits *s_0_* (*i.e.*, *S′[0]*), and hence it is used to encode the entries in *S′* that are smaller than or equal *s_0_*. Recall that *s_0_* is the mean value of the entries in *S_n_*. The larger word is used to encode the entries larger than *s_0_* as it fits the entry with the largest absolute value in *S′*:

(3) $$W(S^{'}\left[i\right])=\;\{\begin{array}{c} \left[\log_{2}S^{'}[0]]\;S^{'}[i]\;\leq S^{'}[0\right]\\ \left[\log_{2}\underset{0\leq k<L}{\max}S^{'}[k])\right]\;S^{'}[i]\;>S^{'}[0] \end{array}$$

To distinguish between these two sizes, a prefix bit is added before each code. A value “0” of this bit indicates the small word size, and “1” for the large word size. Another prefix bit is added for the number's sign.

## 4. Testing Methodology

The three compression schemes explained in the previous section were incorporated in a simulation of a WSN monitoring environment. The monitoring environment contains a set of objects distributed over the simulated monitored area. These objects are monitored through their IDs (unique numbers that identify the monitored objects) and their locations (denoted by using a pair of coordinates X and Y). The objects' monitoring data is collected through a ubiquitous WSN node attached to the object. In addition to the above data, each WSN node reports a stimulus value measured at the object location. That can be any stimulus of interest such as temperature, pressure, or humidity.

The simulation was implemented using Visual C# on an Intel-based PC. The monitoring environment is defined by the following:

- The map of the monitored field (currently defined as a rectangular area).
- The number of monitored objects within the field, which is also the number of WSN nodes (assuming each object is associated with a WSN node).
- The radio range of each WSN node.
- Deployment method of the monitored objects (grid or random deployment).
- The number of areas affected by the stimulus. The simulation allows the stimulus to be allocated randomly or manually by double-clicking on different spots on the field.
- The coverage radius of the stimulus.
- The compression method to be tested (none, Offset, Marcelloni, and LiftingWiSe).
- The dissemination interval after which each WSN node disseminates its collected data to its parent node.

**Figure 4.**
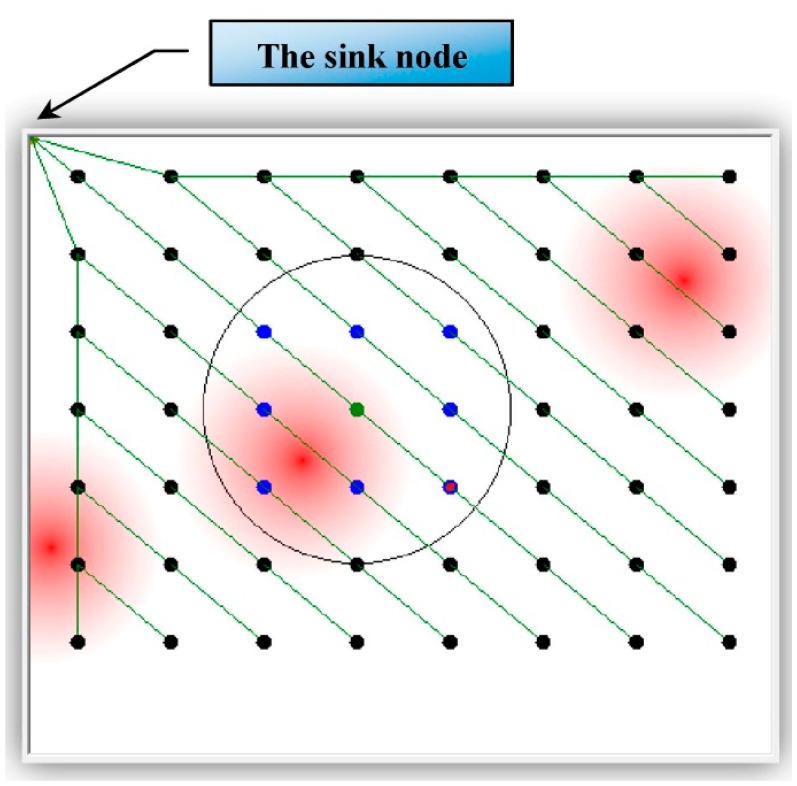
The monitored field with grid-deployed nodes.

**Figure 5.**
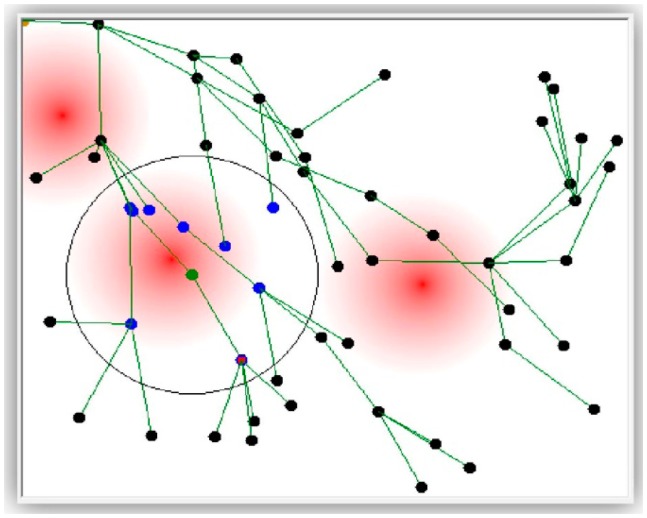
The monitored field with randomly deployed nodes.

Figure 4 shows the simulated monitored field with a grid deployment of 56 WSN nodes. Figure 5 shows the same but with randomly deployed 56 WSN nodes. Both figures show the radio range (a circle) of one of the nodes as well as three areas affected by the stimulus (the darker the red color, the higher the stimulus value).

*Definition 1*: The *sink node* is the node where all disseminated data sent (directly or indirectly) by all nodes in the field are collected. It appears on the top-left corner in the following figures.
*Definition 2*: The neighbors of any node A are those nodes within the radio range of A (appear as blue dots within the circle in the following figures).
*Definition 3*: A parent node is assigned to a set of nodes, the parent's children. A parent is assigned to a child if it is the closest node to the sink from among all the child's neighbors.
*Definition 4*: The basket is the collection of data representing the status of a set of sensor nodes. In the current implementation, the status of a node includes the sensor ID, the sensor location (X and Y coordinates), and the stimulus value at that location.

Each node collects the baskets received from its children, if it has any children, and decompresses them. The decompression process is the reverse process used in the corresponding compression scheme. The node then combines the decompressed baskets in one basket. Note that for the LiftingWiSe scheme, decompression is like compression in terms of all processing steps are done in place (no need for temporary storage for processing). The next step is for the node to add its own status record to the resulted basket (or create a new basket if it does not have one). Finally, the node compresses the content of the resulted basket and disseminates it to its parent. The sink is a special case where it only decompresses the baskets it receives and combines them in one basket. This basket includes the current status records of all sensors in the field. The collected records are eventually forwarded to a server to be analyzed by the field administrator.

The lines connecting the nodes in Figures 4 and 5 are virtual lines representing the dissemination tree. The root of that tree is the sink node. The remaining sensor nodes are represented as vertices of that tree. Each node eventually disseminates its basket to the sink node through its parent in the tree.

*Definition 5*: *Overall disseminated bits* are the total number of bits in the status baskets disseminated by all nodes to their parents until all the data are propagated all the way to the sink.

The implemented simulation has been verified and validated to make sure that it is accurate and credible [33].

### 4.1. Verification

The verification of the simulation is concerned with making sure that the implementation of the model is correct. A dynamic verification was done through testing each function in the simulation with variety of settings of its input parameters. The overall performance of the simulation was stressed tested with WSN fields that have from 100 to 1200 nodes. The nodes were deployed both randomly and as a grid. Stimulus values were randomly allocated on the WSN field. A static verification was achieved through making sure that there are neither inconsistent object declarations nor memory corruptions or leaks.

### 4.2. Validation

The validation of the simulation is concerned with making sure that the implemented model accurately represents the real system. Validation of the simulation's three modules (compression, decompression, and dissemination processes) was achieved through comparing the collected data in the sink node with the given parameters of the deployed nodes. Two fields were graphically drawn on the screen. The first field has all nodes, and stimulus distributed based on the given parameters. The second field contains all nodes, and stimulus displayed based on the collected data at the sink after applying the above three processes. Analyzing both fields under different scenarios validated that the implemented model accurately represents the real system.

## 5. Experimental Results and Analysis

**Figure 6.**
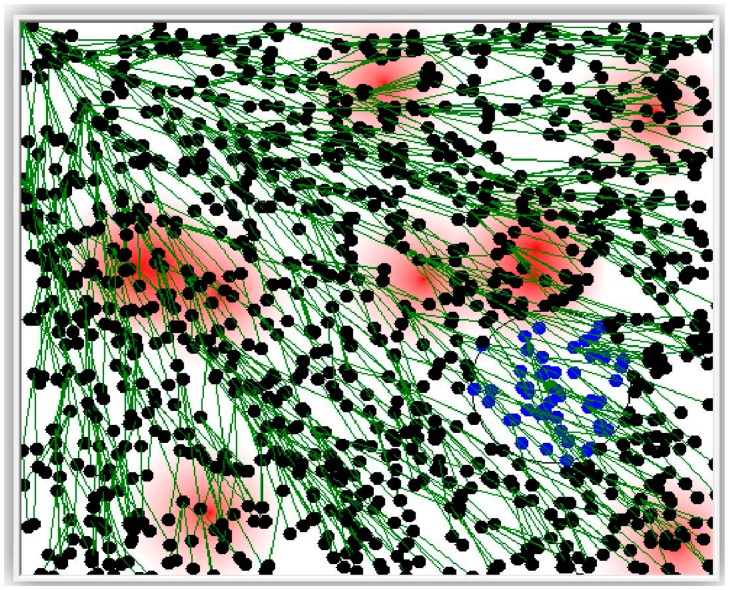
Randomly deployed 1200 nodes.

**Figure 7.**
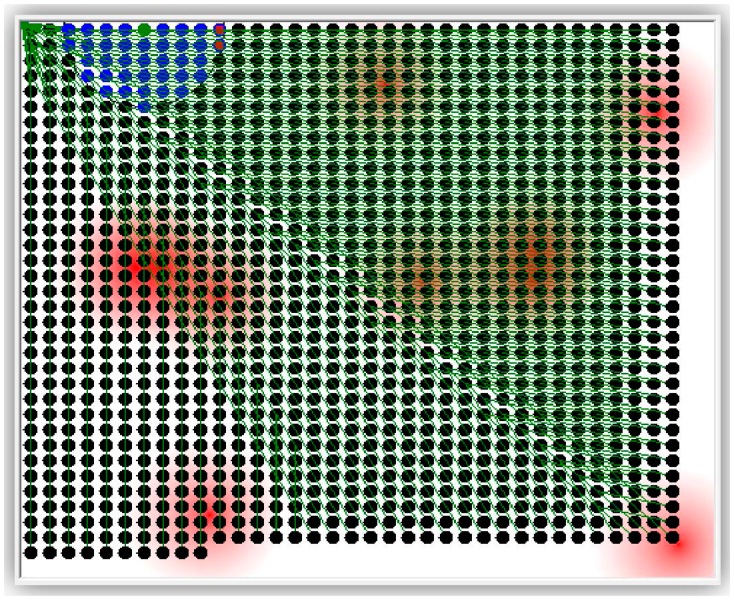
Grid deployment of 1200 nodes.

The simulator has been used to test the following compression techniques: Offset, Marcelloni, and the proposed LiftingWiSe technique. One more run of the simulator was carried out for no compression (None) where sensors' raw data is disseminated without any compression. The overall disseminated bits for each run are recorded and compared. The above compression techniques were tested with 1200 nodes. Both random-deployed nodes (Figure 6) and grid-deployed nodes (Figure 7) were tested.

**Figure 8.**
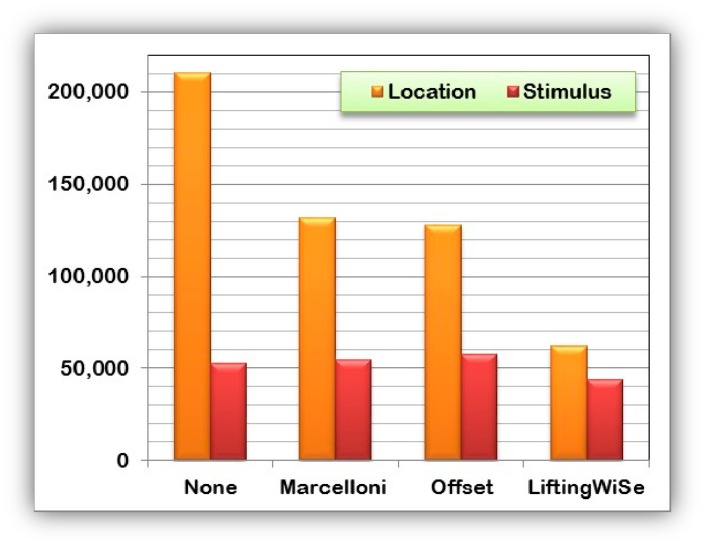
Disseminated bits through 1200 randomly deployed nodes.

**Figure 9.**
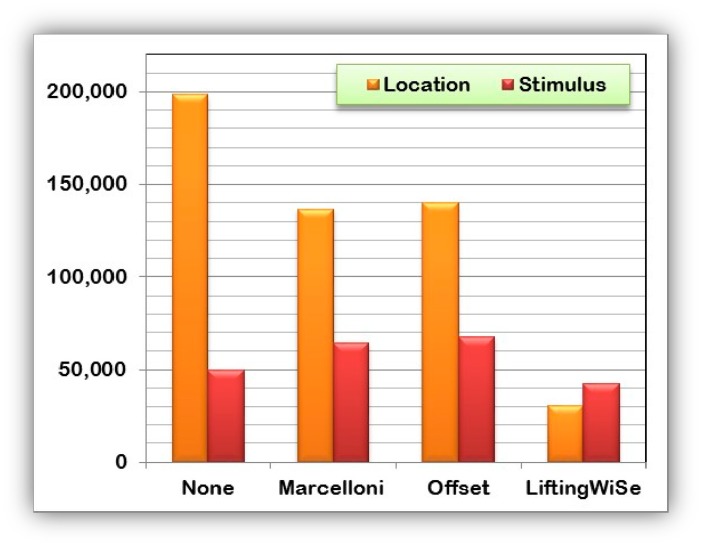
Disseminated bits through 1200 grid deployed nodes.

Figures 8 and 9 compare the performance of the discussed compression techniques. The compression techniques were compared based upon the number of bits disseminated throughout the network until all data is collected at the sink node. Figure 8 shows the comparison for the random deployment while Figure 9 shows the comparison for the grid deployment. For each compression technique, the graphs show the number of bits disseminated to transfer the nodes' locations and the stimulus measured at that location. The following can be observed from the above results:

(1) The proposed LiftingWiSe technique outperforms the other compression techniques for both the locations and stimulus data in both deployment methods.
(2) The locations' data represent a less dynamic signal with large values. However, the stimulus data is a more dynamic signal with small values. Such characteristics explain why disseminating the stimulus signal without data compression (None) performs better than the Offset and Marcelloni techniques.
(3) There is no significant effect of the deployment method on the performance of LiftingWiSe. This is not the case for the Offset and Marcelloni techniques as they are affected by the deployment method.

More simulation runs were conducted to validate the implementation of the simulation. These runs are used to show the following:

(1) The effect of the sensor nodes' radio range on the number of the disseminated bits.
(2) The effect of the sensor nodes' radio range on the execution time of the compression techniques.
(3) The effect of the stimulus coverage over the sensor field on the number of the disseminated bits.

**Figure 10.**
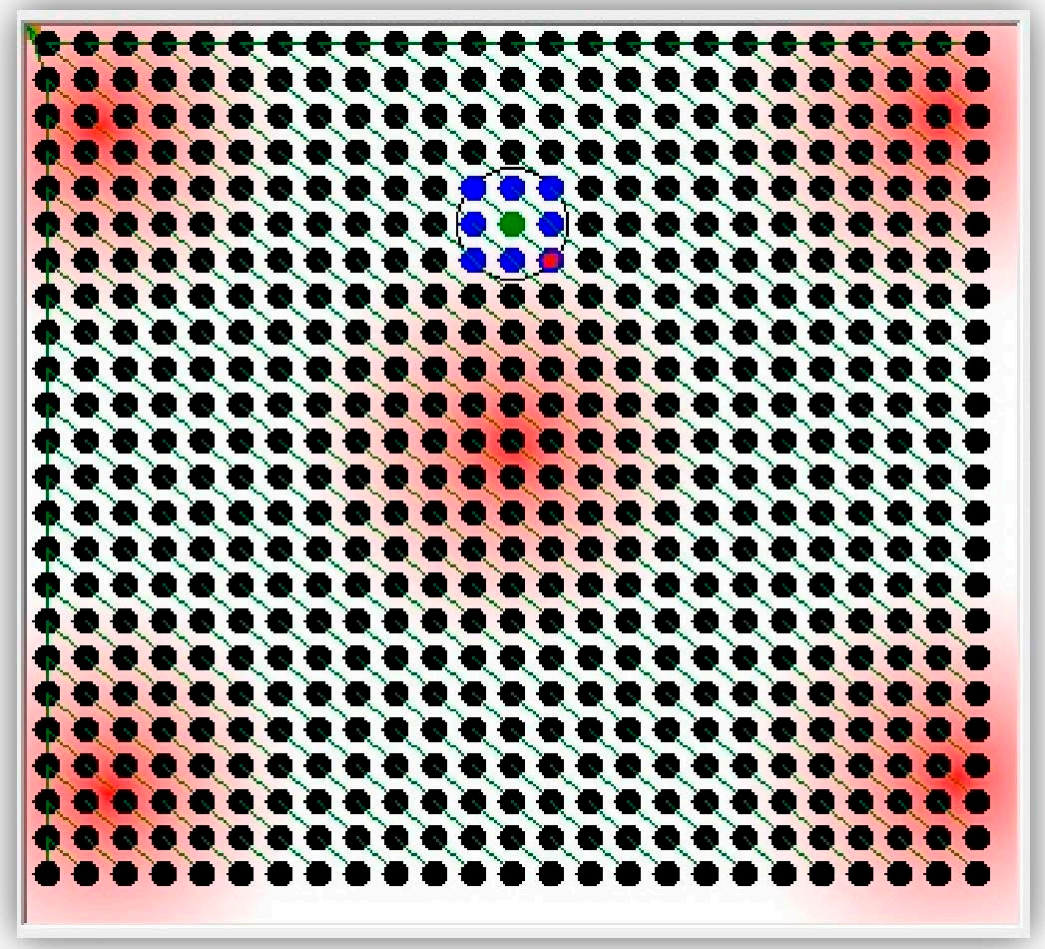
Disseminated bits through 600 grid-deployed nodes.

The grid distribution of 600 deployed sensors used for these runs as shown in Figure 10. The figure shows one settings for the radio range and distribution of the stimulus. The simulation was run for different settings of these parameters.

The radio range in the simulation is measured by its circle diameters in dots. For the grid distribution of Figure 10, the shown radio range covers only the sensor node's closest neighbors. That range has a diameter of 20 dots. LiftingWiSe was tested for radio range diameters from 20 to 200 points, where the 200-point range covers the whole sensor field.

**Figure 11.**
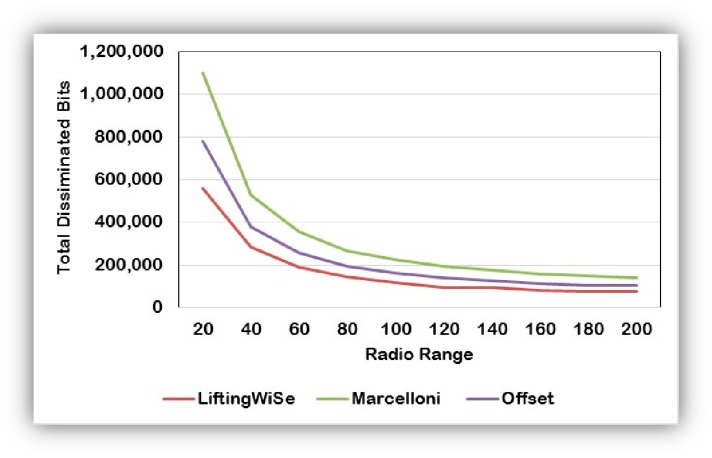
The effect of the radio range on the number of disseminated bits.

Figure 11 shows the effect of the radio range on the total number of bits disseminated throughout a network field with 600 nodes. The graph shows that, for the three studied techniques, the greater the radio range the smaller the number of bits needed to disseminate the nodes' data all the way to the sink node. The reason is that the greater the radio range the less average number of hops on the dissemination paths.

**Figure 12.**
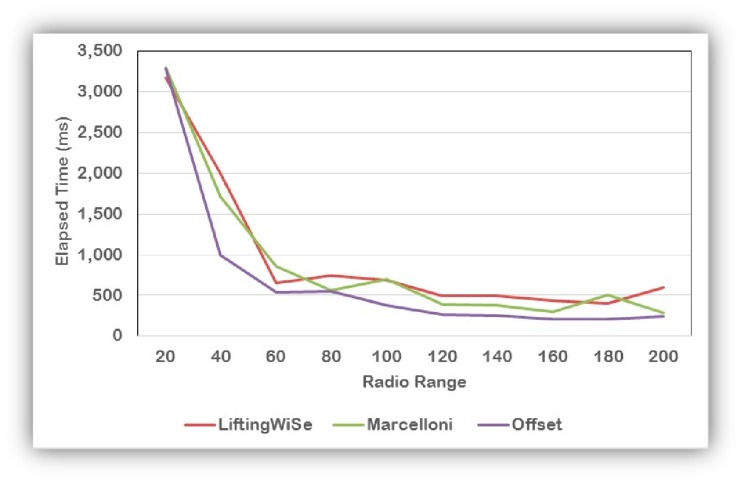
The effect of the radio range on the execution time.

Figure 12 shows the effect of the radio range on the execution time of the studied technique. It shows that as the radio range increases, the execution time decreases. This is because the number of hops needed to reach the sink decreases and hence the compression/decompression processes are executed less frequently.

**Figure 13.**
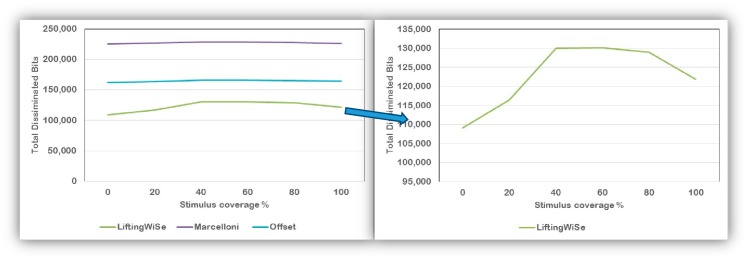
The stimulus coverage effect on the number of disseminated bits.

Figure 13 shows how the number of the disseminated bits are affected by the percentage of the sensor field covered by the stimulus' coverage. A 0% coverage means the field is not affected by the stimulus at all. A 100% coverage means the stimulus covers the whole field with its maximum allowed value. The left graph in Figure 13 shows that both the Offset and Marcelloni techniques are barely affected by the stimulus coverage. The right graph in Figure 13 shows that with LiftingWiSe more bits are generated by the compression technique as the dynamic of the stimulus signal increases. In the extreme cases (*i.e.*, 0% and 100%), the stimulus values are almost constant across the field, which results in better compression rate and less number of disseminated bits.

### Complexity and Integrity Analysis

The complexity and integrity of the proposed LiftingWiSe technique were analyzed. The complexity is measured by calculating the computational complexity [17]. Computational time complexity is proportional to the number of clock cycles required to compress, decompress, and disseminate data throughout a WSN of 1200 nodes. Computational memory complexity is proportional to the memory space needed to perform the above tasks on the data. The integrity of the proposed compression technique is measured by calculating the percent error of the disseminated data compared to its original values.

**Figure 14.**
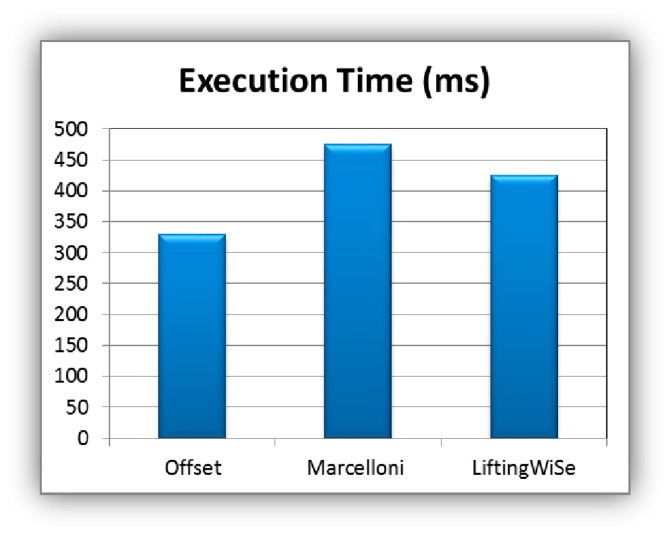
Computational time complexity comparison.

Computational time complexity of LiftingWiSe is measured by the complexity of the lifting wavelet transform and encoding processes that are shown in Figure 3. The computational complexity of both processes is *O(n)*, where n is the length of the signal (*L* in Figure 3). Both the Offset and Marcelloni techniques have computational complexity of *O(n)* as well. Figure 14 shows a quantitative comparison of the execution times of the three compression techniques. The execution time is measured in milliseconds. It represents the required time to disseminate the data throughout the 1200 network nodes. The dissemination process includes data compression, encoding, transmission, decoding, and decompression throughout the network nodes until all data are collected at the sink node. The comparison shows that the proposed LiftingWiSe technique outperforms the Marcelloni technique in terms of execution time. The Offset requires less execution time than the proposed LiftingWiSe technique.

As for computational memory complexity, LiftingWiSe requires less memory space than the Marcelloni technique as LiftingWiSe performs its calculations in place (no need for temporary arrays of data). The Offset technique performs its calculations in place as well. However, LiftingWiSe does not require the overhead of disseminating the reference value with every signal as required by the Offset technique.

As LiftingWiSe is a lossy compression technique, it is important to check for its integrity. Integrity is measured by calculating the percent error of the disseminated data compared to its original values. As for the disseminated X and Y coordinates of all 1200 nodes; the percent error is 0.35%. The percent error is 0.02% for the disseminated stimulus signal. Both percent error values are insignificant considering the nature of the disseminated info. Studies illustrated that the loss due to compression using low-memory wavelet transform techniques is typically not visible [26].

## 6. Conclusions

An efficient and simple data processing technique, LiftingWiSe, for data dissemination in Wireless Sensor Network (WSN) was introduced in this paper. LiftingWiSe is based on the lifting method of the Discrete Wavelet compression technique. LiftingWiSe can be applied on signals with arbitrary length. The algorithm used to implement the compression core of LiftingWiSe has two features. First, it requires simple calculations that do not need a lot of processing power. Second, it performs the compression in place (*i.e.*, one array is needed to store the signal while it is being processed). These features are particularly important due to the limited processing capability and memory space in the WSN nodes. The proposed technique was evaluated on a WSN with 1200 nodes. The nodes were deployed both randomly and in a grid fashion. The disseminated data includes the nodes' locations and the stimulus at those locations. The proposed technique was compared to two other simple compression techniques appropriate to be used in wireless sensor nodes that have very limited processing and memory resources. The results show that LiftingWiSe outperforms the other techniques in terms of the number of bits to be disseminated in the network. Future work includes testing the proposed technique with other types of signals (e.g., multimedia) as well as testing it on a prototype of real sensor nodes.
